# MiR-199a-3p-regulated alveolar macrophage-derived secretory autophagosomes exacerbate lipopolysaccharide-induced acute respiratory distress syndrome

**DOI:** 10.3389/fcimb.2022.1061790

**Published:** 2022-11-29

**Authors:** Xinyi Xu, Xu Liu, Xuecheng Dong, Yi Yang, Ling Liu

**Affiliations:** Jiangsu Provincial Key Laboratory of Critical Care Medicine, Department of Critical Care Medicine, Zhongda Hospital, School of Medicine, Southeast University, Nanjing, China

**Keywords:** secretory autophagosome, alveolar macrophage, miR-199a-3p, RAB8A, PAK4, acute respiratory distress syndrome

## Abstract

**Purpose:**

Acute respiratory distress syndrome (ARDS) is a prevalent illness in intensive care units. Extracellular vesicles and particles released from activated alveolar macrophages (AMs) assist in ARDS lung injury and the inflammatory process through mechanisms that are unclear. This study investigated the role of AM-derived secretory autophagosomes (SAPs) in lung injury and microRNA (MiR)-199a-3p-regulated inflammation associated with ARDS *in vitro* and in a murine model.

**Methods:**

The ARDS model in mouse was established by intratracheal LPS lipopolysaccharide (LPS) injection. The agomirs or antagomirs of MiR-199a-3p were injected into the caudal vein to figure out whether MiR-199a-3p could influence ARDS inflammation and lung injury, whereas the mimics or inhibitors of MiR-199a-3p, siRNA of Rab8a, or PAK4 inhibitor were transfected or applied to RAW264.7 cells to evaluate the mechanism of SAP release. Culture supernatants of RAW264.7 cells treated with LPS or bronchoalveolar lavage fluid from mice were collected for the isolation of SAPs.

**Results:**

We found that MiR-199a-3p was over-expressed in the lungs of ARDS mice. The MiR-199a-3p antagomir alleviated, whereas the MiR-199a-3p agomir exacerbated LPS-induced inflammation in mice by promoting AM-derived SAP secretion. In addition, MiR-199a-3p over-expression exacerbated LPS-induced ARDS *via* activating Rab8a, and Rab8a silencing significantly suppressed the promoting influence of the MiR-199a-3p mimic on SAP secretion. Furthermore, MiR-199a-3p mimic activated Rab8a by directly inhibiting PAK4 expression.

**Conclusion:**

The novel finding of this study is that MiR-199a-3p participated in the regulation of SAP secretion and the inflammatory process *via* targeting of PAK4/Rab8a, and is a potential therapeutic candidate for ARDS treatment.

## Introduction

With morbidity and mortality rates of 10.4% and >40%, respectively, acute respiratory distress syndrome (ARDS) frequently occurs in critically ill patients (Bellani et al., 2016) and is characterized by excessive inflammation, secondary to a variety of pulmonary or extrinsic factors (Calfee, 2015). However, although the mortality remains high, there is currently no valid treatment that modifies the pathogenesis of ARDS.

MicroRNAs (MiRs) belong to a cluster of small, non-coding RNAs that suppress gene expression by attaching to 3′-untranslated regions (UTRs) post-transcriptionally (Chen et al., 2019; Li et al., 2019). Some MiRs contribute significantly to the inflammatory response in ARDS. MiR-199a, a unique one in the MiR family, is involved in several inflammatory responses of immune cells (Rane et al., 2009; Dai et al., 2012) and is associated with inflammatory lung disease, including sepsis-induced ARDS (Liu et al., 2018). However, the molecular and cellular mechanisms that underlie the MiR-based regulation of ARDS lung injury and the inflammatory process are unclear.

Nearly half of the immune cells in the lung are macrophages, and these contribute significantly to the pathogenesis of ARDS by synthesizing and releasing various inflammatory mediators (Hashimoto et al., 2013; Deprez et al., 2020; Jardine and Haniffa, 2021). There is emerging evidence of the crucial role of extracellular vesicles and particles from alveolar macrophages in the progression of the inflammatory process of ARDS. Previous studies demonstrated that alveolar macrophages contribute the majority of extracellular vesicles (EVs) and particles in the bronchoalveolar lavage fluid (BALF) from mice with early-stage LPS-stimulated ARDS (Soni et al., 2016; O'Dea et al., 2020; Hu et al., 2021). Moreover, it has been shown that EVs and particles derived from macrophages contain proinflammatory mediators, and lipid fractions of EV membranes have proinflammatory properties (Thomas and Salter, 2010; Wang et al., 2011; Lee et al., 2011; Islam et al., 2012). We previously identified a novel type of vesicles from AMs, which could be characterized as double-membrane vesicles with the expression of light chain 3 (LC3) and which could be considered as secretory autophagosomes (SAPs). We found that AM-derived secretory autophagosomes could amplify the inflammation and aggravate the lung injury of ARDS, and therefore regulating SAP secretion would significantly alleviate the inflammatory and pathological injury of ARDS (Xu et al., 2022). However, the participation of microRNAs in regulating AM-derived SAP secretion remains to be clearly defined. Therefore, we evaluated the impact of MiR-199a-3p and its potential mechanism in AM-derived SAP secretion and inflammation in ARDS.

## Material and methods

### Animal procedures

Male C57BL/6 mice (8–10 weeks) were applied in experiments, which was approved by the Committee of Animal Care and Use of Southeast University (Protocol number 20180106007). Mice (n = 5–7 per group) were administered with lipopolysaccharide (5 μg/g; MCE, USA) *via* intratracheal injection to create an ARDS model. Agomir, agomir NC (dose: 5 nmol/mouse), antagomir, or antagomir NC of MiR-199a-3p (dose: 50 nmol/mouse) was administered by caudal vein injection and followed by the establishment of the ARDS model 24 h after this administration. Normal saline (NS) was applied as the negative control and the vehicle for LPS administration. After 24 hours, bronchoalveolar lavage fluid (BALF) was acquired. Lung tissue from the mice was collected for HE staining, and the levels of pro-inflammatory cytokines of lung tissue were measured with an enzyme-linked immunosorbent assay (ELISA) kit (Ela Science, People’s Republic of China), which included TNF-α, IL-12, IL-10, and IL-6. We also conducted *in vitro* experiments to mimic an *in vivo* environment to preclude the effect of endogenous RNase/DNase on agomir and antagomir in this study (**Figure S1**).

### Cell culture

RAW264.7 cells were acquired from the Cells Resource Center for Biological Science (Shanghai, China). RAW264.7 cells were maintained in a DMEM medium (Wisent Biotechnology, China); 10% fetal bovine serum and 1% penicillin-streptomycin were applied, and we cultured the cells at 37°C in an incubator with 5% CO_2_.To assess the influence of lipopolysaccharide-stimulated on activating RAW264.7 cells, two groups of cells were treated with phosphate-balanced solution or LPS (MCE, USA) at different times. To ensure PAK4 inhibition by pretreatment, we add PF3758309 (20 nM; Sigma) into the medium 24 h before the administration of LPS. Mouse AMs were isolated using previously described protocol (Soni et al., 2016).

### Cell viability analysis

To assess the cytotoxic influence of lipopolysaccharide on macrophages, we conducted the CCK-8 assay and trypan blue exclusion assay. The cells were seeded into 96-well plates (2000 cells/100 µl), incubated at 37°C overnight, and then treated with 0, 10, 100, or 1000 ng/ml LPS for 24 h. Then, 10 µl CCK8 (Beyotime, China) was added to each well, and the cells were incubated for 4 h. Afterwards, the absorbance of the cells at 450 nm was measured using a microplate reader. We also conducted the trypan blue exclusion assay to evaluate the cytotoxic effect of LPS. After administration with LPS of different concentrations for 24 h, the viability of RAW364.7 cells was evaluated by counting the number of viable cells.

### RNA interference and cell transfection

The negative controls (NC), mimics, or inhibitors of MiR-199a-3p (50 nM; Genepharma, Shanghai) were transfected into the RAW264.7 cells based on the manufacturer’s procedure. RAW264.7 cells were seeded into a 6-cm dish at a density of 1 × 10^5^/ml. The transfection was performed by adding PoweverFectTM transfection reagents (SignaGen, USA) with MiR-199a-3p mimic, MiR-199a-3p inhibitor, or NC. The optimal concentration of the transfection reagent was determined by detecting the intensity of green fluorescence generated in the transfected cells. After the transfection, the cells should be washed by phosphate-balanced solution for three times. After 48 h, the medium ought to be replaced, and the efficacy of transfection was detected by RT-qPCR. On the other hand, RAW264.7 cells were also transfected by the RNAi of Rab8a (RiboBio, Guangzhu), pursuant to the manufacturer’s procedure. The efficiency was measured by Western blotting analysis. Moreover, after transfection, we conducted the CCK8 assay to assess the cytotoxic influence of siRNA, mimics, inhibitors, and negative controls (**Figure S2**).

### Isolation of secretory autophagosomes

We collected secretory autophagosomes in accordance with the methods published previously (Xu et al., 2022). In order to remove debris, the cell culture supernatants or bronchoalveolar lavage fluid samples were acquired and centrifuged at 400×*g* for 5 min at first; then, we collected the supernatants for centrifugation of 12,000×*g* for 30 min. After that, the supernatants were removed, and then we washed the SAP-containing pellets with phosphate-balanced solution for three times. Subsequently, the pellets were purified with LC3b antibody attained magnetic beads (Miltenyi Biotec; Cell Signaling Technology). Finally, we resuspended the acquired pellets in phosphate-balanced solution and stored them in a −80°C refrigerator for further experiments.

### Nanoparticle tracking analysis

We tested concentrations and sizes of secretory autophagosomes by NTA with ZetaView PMX (Particle Metrix, Germany). First, we diluted the samples with 1× phosphate-balanced solution. Then, NTA measurements were conducted at 11 different positions; 110 nm polystyrene particles were applied for calibration of the ZetaView system. The temperature should be maintained from 23°C to 30°C.

### Transmission electron microscopy

Firstly, we deposited the sample on the formvar-carbon grid and hatched it at room temperature for 5 min. Next, 1% uranyl acetate was added for 5 s. Then, we put three drops of water on the parafilm to wash the grid for three times. When the grid had dried, we applied the transmission electron microscope to observe the sample at 80 kV.

### Western blotting analysis

We applied the RIPA lysis buffer to obtain proteins from cell lysates and SAPs. The concentrations of protein were determined by the bicinchoninic acid (BCA) protein assay (Beyotime, China). Protein was separated by 10%–15% SDS-PAGE gel for electrophoresis and transferred to PVDF membranes (Bio-Rad, American). Then, we blocked the membranes with 5% bovine serum albumin (BSA) for 1 h at room temperature and hatched with primary antibodies overnight at 4°C, which included antibodies against F4/80, LC3B, β-actin, Rab8a (1:1000; #6975, #43566,#4970 and #30325, CST), and PAK4 (1:1000, pS474, Absin). Subsequently, the membranes were then hatched with the horseradish peroxidase-conjugated secondary antibody (1:5000; Beyotime, China) for 1 h at 4°C and then the protein bands were visualized with the electrochemiluminescence detection kits (Beyotime, China).

### ELISA

The levels of pro-inflammatory cytokines were quantified by ELISA kit pursuant to the manufacturer’s protocols (Ela Science, China), which included IL-6, IL-12, TNF-α, IL-10, and IL-1β. We applied the microplate reader to acquire the OD450 values of each sample.

### Real-time quantitative polymerase chain reaction

We used TRIzol^®^ (Takara, Japan) to acquire total and quantified RNA with a NanoDrop ND-2100 spectrophotometer (Thermo Scientific). Then, the RNA was reversely transcribed with the Transcriptor First Strand cDNA kit (Ribobio), and U6 was applied as the internal control. Subsequently, we performed the qPCR with specific primers (Ribobio), and the primer sequences included: MiR-199a-3p, 5′-CAGTGTTCAGACTACCTGTTC-3′; U6: 5′-GTGCTCGCTTCGGCAGCACATAT-3′. Afterwards, we conducted qPCR amplification with SYBR Green PCR Master Mix (Ribobio) on a LightCycler 96 system (Roche, Switzerland).

### Dual luciferase reporter plasmid assay

HEK293 cells were used to conduct the luciferase reporter assay. The cells were cotransfected with the reporter plasmids and MiR-199a-3p mimics or MiR-NC plasmids with either the wild-type or mutant PAK4 3′-UTR (Promega, USA) using the Lipofectamine 2000 reagent (Invitrogen, USA). Then, after 24 h, luciferase activity was measured with a luciferase analysis reagent kit, pursuant to the manufacturer’s instructions (Promega).

### Statistical analysis

All experiments in this study were repeated for at least three times. We applied GraphPad Prism 8.0 (GraphPad Software, USA) to conduct data analysis. Results were signified as the mean ± standard deviation (SD) and compared by using Student’s *t*-test or one-way analysis of variance (ANOVA) with a cutoff of p < 0.05. Kaplan-Meier curves were used to demonstrate survival rates, while survival curves were compared using the log-rank test.

## Results

### LPS-stimulated inflammatory reaction and MiR-199a-3p expression

**Figure 1:**
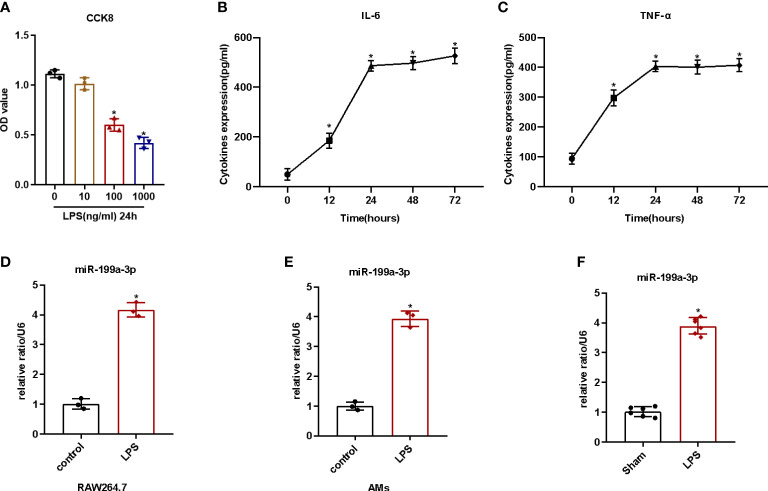
LPS-stimulated inflammatory reaction and MiR-199a-3p expression *in vivo* and *in vitro*. RAW264.7 cells were stimulated with or without LPS for 12, 24, 48, and 72 h. **(A)** The effects of various concentrations of LPS on the viability of RAW264.7 cells after 24 h treatment, as tested *via* CCK-8 assay (n = 3). **(B)** The effect of LPS (100 ng/ml) on the level of IL-6 in cell supernatants, as tested *via* ELISA (n = 3). **(C)** The effect of LPS (100 ng/ml) on the level of TNF-α in cell supernatants, as tested *via* ELISA (n = 3). **(D)** qRT-PCR detection of MiR-199a-3p expression in RAW264.7 cells after LPS treatment (n = 3). **(E)** qRT-PCR detection of MiR-199a-3p expression in alveolar macrophages after LPS treatment (n = 3). **(F)** MiR-199a-3p expression in BALF of LPS-induced ARDS or control mice was detected by qRT-PCR (n = 6). The experiments were repeated at least three times. Each value represents the mean ± SD of three independent experiments. *p < 0.05 *vs*. control group, Student’s *t*-test, or one-way ANOVA.

First, we detected the cell viability after LPS administration in order to identify the cytotoxic influence of lipopolysaccharide on RAW264.7. We found that the cell viability of RAW264.7 cells decreased significantly with 100 ng/ml LPS (**Figures 1A**, **S3**). Then, we added 100 ng/ml LPS to the medium at different times to evaluate the influence of LPS on activating RAW264.7 cells. The results illustrated that lipopolysaccharide treatment for 24 h could increase the levels of the pro-inflammatory cytokines (IL-1β and TNF-α) of the cell supernatants remarkably (**Figures 1B, C**). Therefore, we considered 100 ng/ml LPS induction for 24 h as an adequate condition to provoke inflammation in RAW264.7 cells. Additionally, we noticed that the expression of MiR-199a-3p in both AMs and RAW264.7 cells increased significantly after LPS administration (**Figures 1D, E**). Furthermore, we quantified MiR-199a-3p expression in the BALF of mice with LPS-induced ARDS. Our results pointed out that, compared with control group, the MiR-199a-3p expression level in the BALF significantly increased (**Figure 1F**). Therefore, MiR-199a-3p expression increased after LPS administration, both *in vitro* and *in vivo*.

### MiR-199a-3p over-expression exacerbated LPS-induced ARDS in mice

**Figure 2:**
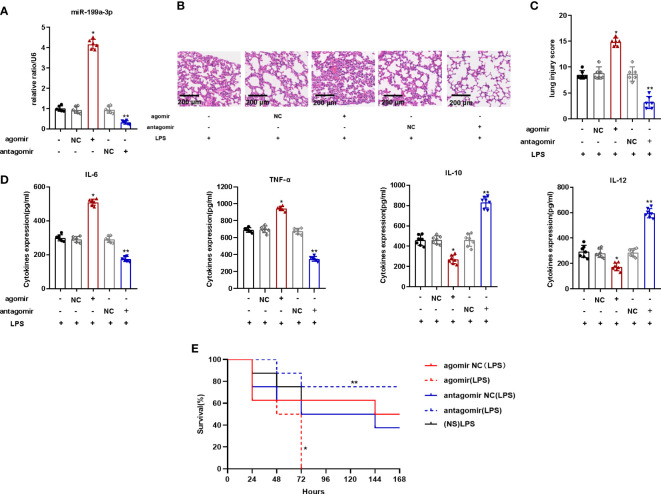
MiR-199a-3p over-expression exacerbated LPS-induced ARDS in mice. The agomir of MiR-199a-3p, agomir NC, antagomir of MiR-199a-3p, and antagomir NC were injected into mice *via* the tail vein. **(A)** MiR-199a-3p expression in lung tissue from mice in each group was detected by qRT-PCR (n=6 per group). **(B)** Histopathological images of lung samples (haematoxylin and eosin staining; scale bar = 200 µm). **(C)** Injury scores of the lung samples (n=6 per group). **(D)** Inflammatory cytokine levels in the lungs of mice from different groups were assessed using ELISA (n=6 per group). **(E)** Kaplan-Meier survival curves were used to demonstrate the effect of MiR-199a-3p on murine survival (n=7 per group). The experiments were repeated at least three times. Each value represents the mean ± SD of three independent experiments. *p < 0.05 *vs*. the agomir NC group, **p < 0.05 *vs*. the antagomir NC group, one-way ANOVA or log-rank test.

To explore the impact of MiR-199a-3p in mice with ARDS, we injected the MiR-199a-3p agomir or antagomir into mice *via* the caudal vein before the establishment of the ARDS model by intratracheal LPS administration. The MiR-199a-3p expression in the BALF of mice with agomir increased significantly (**Figure 2A**). Compared to mice of control group, over-expression of MiR-199a-3p aggravated the pathological injury of lung tissues (**Figures 2B, C**). The results of ELISA demonstrated that proinflammatory cytokine (TNF-α and IL-6) levels increased after agomir administration, while the levels of anti-inflammatory cytokines (IL-10 and IL-12) decreased significantly (**Figure 2D**). In addition, compared with the NC groups, the mortality of mice injected with agomir increased remarkably (**Figure 2E**). Thus, MiR-199a-3p over-expression probably aggravates LPS-stimulated ARDS in mice.

### MiR-199a-3p overexpression increased SAP secretion of mice in ARDS

**Figure 3:**
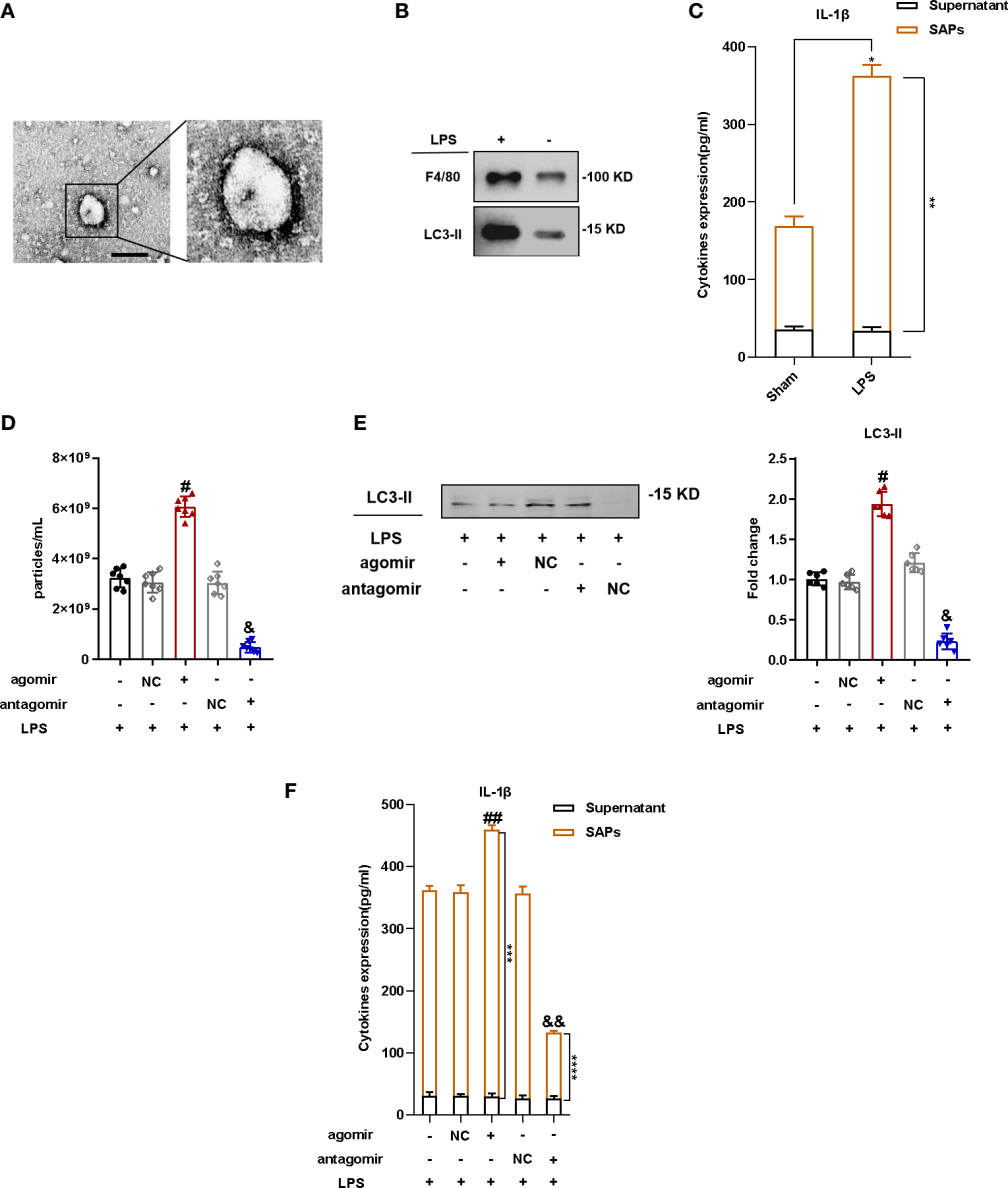
MiR-199a-3p over-expression increased SAP secretion in ARDS mice. SAPs were isolated from the BALF of mice *via* centrifugation. **(A)** The vesicles from BALF were identified *via* transmission electron microscopy (scale bar 200 nm). **(B)** Western blotting analysis revealed that the vesicles from BALF expressed LC3-II and F4/80. **(C)** The levels of IL-1β in SAPs and BALF supernatant were assessed *via* ELISA after the SAPs were lysed using Triton X-100 (n = 6 per group). **(D)** Nanoparticle tracking analysis revealed significant increases in the levels of vesicles in the BALF of mice injected with the agomir of MiR-199a-3p, compared with the control group (n = 7 per group). **(E)** Western blot analysis demonstrated significant increases in the levels of LC3-II in vesicles from the BALF of mice injected with the agomir of MiR-199a-3p, compared with the control group (n = 7 per group). **(F)** The levels of IL-1β in SAPs and the BALF supernatant were assessed *via* ELISA after the SAPs were lysed using Triton X-100 (n = 6 per group). The experiments were repeated at least three times. Each value represents the mean ± SD of three independent experiments. *p < 0.05 *vs*. the SAPs + supernatant in the sham group; **p < 0.05 *vs*. the SAPs in the sham group; #p < 0.05 *vs*. agomir NC group; & p <0.05 *vs*. antagomir NC group; ##p< 0.05 *vs*. the SAPs + supernatant in the agomir NC group; ***p < 0.05 *vs*. the SAPs in the agomir NC group; && p < 0.05 *vs*. the SAPs + supernatant in the antagomir NC group; ****p < 0.05 *vs*. the SAPs in the antagomir NC group, Student’s *t*-test or one-way ANOVA.

To investigate the mechanism of lung injury exacerbation with MiR-199a-3p overexpression, we isolated vesicles from the BALF of mice and verified the double-membrane structure with TEM (**Figures 3A**, **S4**). We also conducted NTA to assess the size of vesicles from BALF, which was consistent with that of autophagosomes (**Figure S5**). The marker of autophagosome LC3 and the marker of macrophages F4/80 was detected by Western blotting (**Figure 3B**), and this indicated that the vesicles isolated from BALF could be considered as SAPs, which were at least partially released from macrophages. As the proinflammatory cytokine IL-1β was the vital molecule inside SAPs that caused lung injury in mice (Soni et al., 2016), we applied the ELISA kit to test the level of IL-1β in secretory autophagosomes from BALF after breaking the SAP membrane with Triton X-100. These results indicated much more IL-1β in secretory autophagosomes from the BALF of LPS-induced ARDS mice than those in the control group (**Figure 3C**). Following the injection of agomir, agomir-NC, antagomir, and antagomir-NC of MiR-199a-3p, the results of NTA revealed that the quantity of particles increased significantly in bronchoalveolar lavage fluid obtained from mice in ARDS with MiR-199a-3p overexpression against that in the NC group (**Figure 3D**). The results of Western blotting illustrated that LC3 was much more sufficient in the BALF from mice with MiR-199a-3p overexpression (**Figure 3E**). Furthermore, IL-1β levels in BALF noticeably increased with MiR-199a-3p over-expression, especially for SAPs in BALF (**Figure 3F**). Therefore, MiR-199a-3p over-expression markedly promotes SAP release in mice with ARDS. Therefore, MiR-199a-3p over-expression markedly promotes SAP release in mice with ARDS.

### MiR-199a-3p overexpression promoted SAP release in LPS-stimulated macrophages

**Figure 4:**
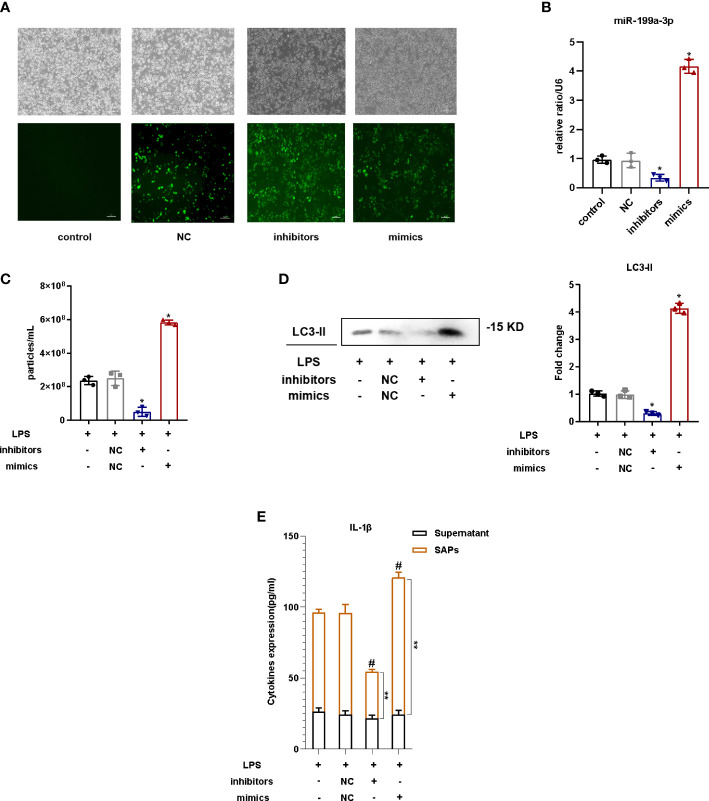
MiR-199a-3p overexpression stimulated SAP secretion in LPS-induced macrophages. Before LPS treatment, RAW264.7 cells were transfected with MiR-199a-3p mimics or inhibitors. **(A)** The efficacy of transfection was detected by fluorescence microscopy. **(B)** MiR-199a-3p levels were examined after 24 h of transfection using qRT-PCR. **(C)** Nanoparticle tracking analysis revealed significant increases in the levels of vesicles from LPS-stimulated RAW264.7 cells in the MiR-199a-3p mimics groups compared with those in the control groups. **(D)** Western blotting analysis demonstrated that there were significant increases in the levels of LC3-II in vesicles from LPS-stimulated RAW264.7 cells in the MiR-199a-3p mimics groups compared with those in the control groups. **(E)** IL-1β levels in SAPs and cell-culture supernatant were assessed *via* ELISA after the SAPs were lysed using Triton X-100. The experiments were repeated at least three times (n = 3 per group). Each value represents the mean ± SD of three independent experiments. *p < 0.05 *vs*. NC group; #p < 0.05 *vs*. the SAPs + supernatant in the NC group; **p < 0.05 *vs*. the SAPs in the NC group, one-way ANOVA.

To explore the influence of MiR-199a-3p on the SAP release from LPS-provoked RAW264.7 cells, cells were transfected with the MiR-199a-3p mimic or inhibitor before LPS administration, and the efficiency was determined by fluorescence microscopy and qRT-PCR (**Figures 4A, B**). The results of NTA demonstrated that the quantity of vesicles increased prominently in the supernatant of RAW264.7 cells with MiR-199a-3p overexpression compared with that in the NC group (**Figure 4C**). Western blotting analysis revealed that the level of LC3 was much more sufficient in the RAW264.7 cell supernatant with MiR-199a-3p overexpression (**Figure 4D**). Additionally, IL-1β levels in SAPs markedly increased with MiR-199a-3p mimic transfection (**Figure 4E**). Therefore, SAP secretion from macrophages could be induced by MiR-199a-3p over-expression.

### MiR-199a-3p overexpression enhanced SAP secretion *via* Rab8a activation *in vitro*

**Figure 5:**
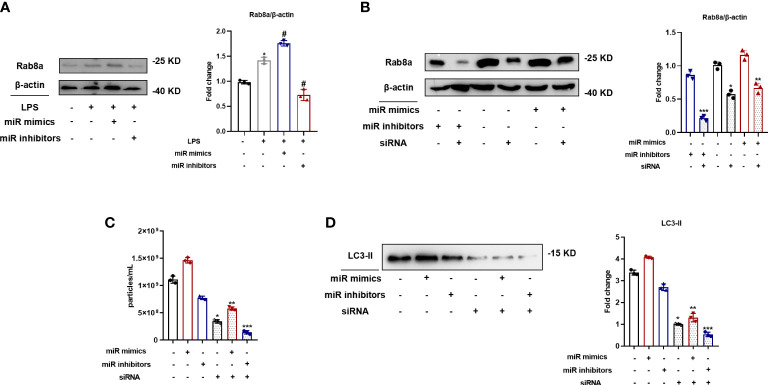
MiR-199a-3p mimics augmented LPS-induced SAP secretion *via* the activation of Rab8a *in vitro*. **(A)** Western blot analysis demonstrated the Rab8a levels in RAW264.7 cells with the transfection of MiR-199a-3p mimics or inhibitors. **(B)** Western blot analysis illustrated the efficient siRNA-mediated knockdown of Rab8a in RAW264.7 cells with the transfection of MiR-199a-3p mimics or inhibitors. **(C)** NTA revealed significant decreases in vesicle levels from RAW264.7 cells with Rab8a knockdown in the MiR-199a-3p mimic and inhibitor groups compared with the control group. **(D)** Western blot analysis demonstrated significant decreases in LC3-II levels in vesicles from LPS-stimulated RAW264.7 cells in the Rab8a knockdown groups compared with the control group. The experiments were repeated at least three times (n = 3 per group). Each value represents the mean ± SD of three independent experiments. *p < 0.05 *vs*. the control; #p < 0.05 *vs*. the LPS control; **p < 0.05 *vs*. the mimics group without Rab8a knockdown; ***p < 0.05 *vs*. the inhibitors group without Rab8a knockdown, one-way ANOVA.

To ascertain the relevant signaling pathways involved in MiR-199a-3p-regulated SAP secretion, Rab8a pathways were investigated because of the vital role in SAP secretion, as determined in our previous study (Soni et al., 2016). The expression levels of Rab8a increased after the transfection of the MiR-199a-3p mimic in RAW264.7 cells (**Figure 5A**). In addition, Rab8a in RAW264.7 cells transfected with the MiR-199a-3p mimic or inhibitor was knocked down by siRNA, and the efficiency was validated by Western blot analysis (**Figure 5B**). Both NTA and Western blot analysis demonstrated that Rab8a silencing significantly inhibited the secretion of SAPs (**Figures 5C, D**). Notably, Rab8a silencing reversed the promoted influence of the MiR-199a-3p mimics on SAP secretion in LPS-stimulated macrophages. Therefore, these results imply that MiR-199a-3p mimics stimulate LPS-induced SAP secretion *via* the activation of Rab8a *in vitro*.

### MiR-199a-3p overexpression activated Rab8a by directly reducing PAK4 expression

**Figure 6:**
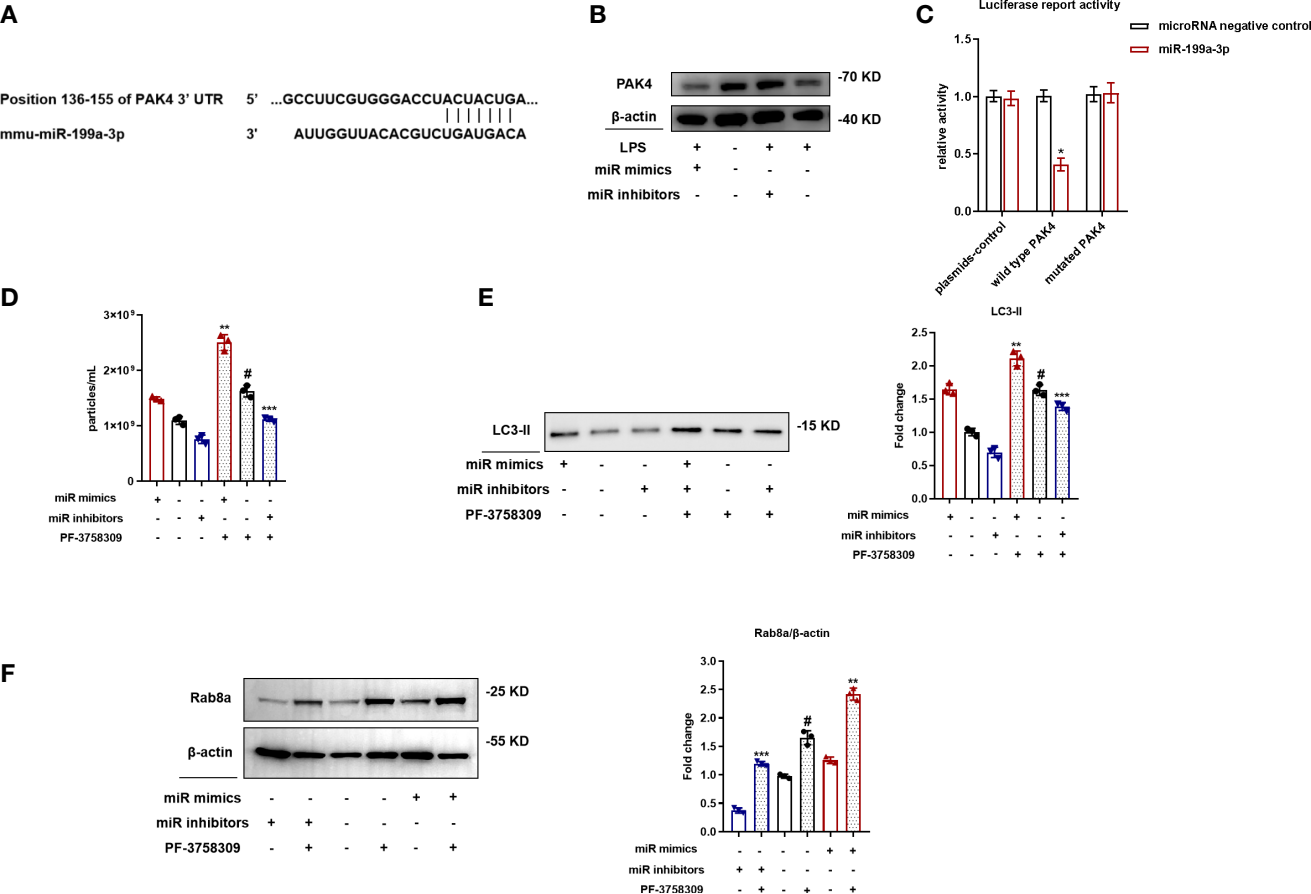
MiR-199a-3p mimics activate Rab8a by directly reducing PAK4 expression. **(A)** The predicted MiR-199a-3p binding site on the PAK4 3′-UTRs was determined using target prediction software. **(B)** Western blot analysis validated the PAK4 expression when cells were transfected with the MiR-199a-3p mimic/inhibitor. **(C)** Luciferase reporter plasmid assays with wild-type and mutated PAK4 plasmids co-transfected with MiR-199a-3p- or MiR-NC-packaged plasmids. Dual luciferase control vector plasmids acted as NCs. **(D)** Nanoparticle tracking analysis revealed significant increases in the level of vesicles from PF-3758309-stimulated RAW264.7 cells in the MiR-199a-3p mimic and inhibitor groups compared with the control group. **(E)** Western blot analysis demonstrated significant increases in LC3-II levels in vesicles from PF-3758309-stimulated RAW264.7 cells in the MiR-199a-3p mimic and inhibitor groups compared with the control group. **(F)** Western blot analysis revealed the effect of PF-3758309 on increasing Rab8a levels in RAW264.7 cells transfected with MiR-199a-3p mimics and inhibitors. The experiments were repeated at least three times (n = 3 per group). Each value represents the mean ± SD of three independent experiments. *p < 0.05 *vs*. the plasmid-control group transfected with MiR-199a-3p plasmids; #p < 0.05 *vs*. the control group; **p < 0.05 *vs*. the mimics group without PF-3758309; ***p < 0.05 *vs*. the inhibitors group without PF-3758309, one-way ANOVA.

Rab8a is a small guanosine triphosphatase (GTPase) that belongs to the Ras superfamily, which could be regulated by PAK4. We used TargetScan to recognize latent target genes of MiR-199a-3p. According to assumed target sequences at positions 136–155 of the PAK4 3′-UTR (**Figure 6A**), PAK4 was considered as a latent target of MiR-199a-3p. Western blot analysis showed that MiR-199a-3p mimics suppressed the expression of PAK4 in RAW264.7 cells (**Figure 6B**). Next, a luciferase reporter analysis showed that MiR-199a-3p repressed the luciferase activity of cells transfected with wild-type PAK4 3′-UTRs, but did not change the luciferase activity of cells transfected with mutated PAK4 3′-UTRs (**Figure 6C**). These results confirm that PAK4 is a direct target for MiR-199a-3p. To clarify the involvement of PAK4 in SAP secretion, we inhibited PAK4 expression by using the PAK4 inhibitor PF-3758309 in RAW264.7 cells transfected with MiR-199a-3p mimic or inhibitor. The results demonstrated that PAK4 inhibition with PF-3758309 in RAW264.7 cells transfected with the MiR-199a-3p mimic or inhibitor significantly promoted SAP secretion. Additionally, MiR-199a-3p mimics, combined with PAK4 inhibition, synergistically enhanced SAP secretion, compared with other groups (**Figures 6D, E**). Furthermore, MiR-199a-3p inhibitors markedly suppressed Rab8a expression in LPS-induced macrophages, yet failed to suppress Rab8a expression after PAK4 inhibition (**Figure 6F**). These findings indicate that MiR-199a-3p over-expression activates Rab8a by directly decreasing PAK4 expression.

## Discussion

Our study is the first to elucidate that MiR-199a-3p plays a crucial role in the pathogenesis of ARDS by promoting the secretion of AM-derived SAPs, which could stimulate the inflammatory process and thus exacerbate lung injury. The insights from this study could illuminate new therapeutic targets to alleviate inflammation in ARDS by reducing SAP secretion. Moreover, this study uncovered a new understanding of the mechanism of miRNA-regulated SAP secretion. Our findings demonstrate that the PAK4/Rab8a pathway is essential for the SAP secretion of AMs and could be modulated by MiR-199a-3p, which may be a latent curative candidate for ARDS treatment.

Increasingly, evidence demonstrates that specific genes contribute significantly to ARDS pathogenesis and prognosis. Among the gene regulators discovered so far, miRNAs are possible mediators of ARDS progression (Rubenfeld et al., 2005; Oglesby et al., 2010; Wang et al., 2016). For example, MiR-16 could suppress NLRP3 inflammasome activation by targeting Toll-like receptor 4 directly in acute lung injury (ALI) (Yang et al., 2019). Li et al. also showed that MiR-33 is involved in acute lung injury provoked by LPS, too (Li et al., 2019). Moreover, over-expression of MiR-146b could alleviate ARDS, both in mice and in cells (He et al., 2019). MiR-199a-3p belongs to a polymorphous MiR family, which is associated with multifactorial lung inflammation. Recently, some studies have illustrated that several pro-inflammatory cytokines are modulated by MiR-199a (Rane et al., 2009; Zuo et al., 2016). In our study, the MiR-199a-3p antagomir attenuated LPS-induced inflammatory responses, which was evident from downregulated secretion of pro-inflammatory cytokine secretion; alleviated lung injury; and decreased mortality in ARDS mice. Notably, the MiR-199a-3p antagomir significantly inhibited SAP release and IL-1β expression in BALF from mice with LPS-induced ARDS. The production of pro-inflammatory cytokines is an important feature of ARDS, and therapeutic targeting of these cytokines can ameliorate ARDS progression (Rubenfeld et al., 2005; Vanden Berghe et al., 2014). We demonstrated that AM-derived SAPs contributed to the overwhelming inflammation of ARDS *via* secretion of IL-1β (Xu et al., 2022). Thus, it is reasonable to assume that the MiR-199a-3p antagomir could have a protective function by modulating SAP secretion, which implies that the MiR-199a-3p antagomir may be a curative candidate for the treatment of ARDS.

Furthermore, MiRs could attach to the 3′-UTRs region of target mRNA to modulate gene expression post-transcriptionally, which can lead to the translational suppression or demotion of target mRNAs. PAK4 is one of the subfamilies of serine/threonine kinase, named group II PAKs, which are widely expressed (Jin et al., 2015). A previous study demonstrated that PAK4 could be a direct target of MiR-199a-3p in hepatocellular carcinoma (Callegari et al., 2018). Moreover, PAK4 was identified as a key regulator of TNF-induced endothelial cell-derived microparticle release, which suggested that PAK4 was involved in the regulation of vesicular secretion (Zhang et al., 2020). Thus, PAK4 may mediate SAP release from AMs in ARDS. In this study, by the online prediction algorithm TargetScan, we found that PAK4 could be a direct target for MiR-199a-3p, and we verified this by dual luciferase reporter analysis. Our results suggested that MiR-199a-3p reduced the dual luciferase activity significantly in cells transfected with the wild-type PAK4 3′-UTR but not with the mutant PAK4 3′-UTR. Meanwhile, Western blotting analysis indicated that PAK4 expression in RAW264.7 cell lines was downregulated remarkably by MiR-199a-3p mimics. Moreover, pretreatment with the PAK4 inhibitor (PF-3758309) reversed the negative effect of MiR-199a-3p inhibitors on the SAP secretion from LPS-induced macrophages. These results illustrate that MiR-199a-3p modulates SAP secretion by PAK4 degradation, which proves that PAK4 could be the target gene for MiR-199a-3p in ARDS.

A previous study demonstrated that PAK4 plays a key role in Ras signaling, which contributes to the aggressive malignant phenotypes of rhabdomyosarcoma (Dasgupta et al., 2021). The Ras superfamily of small GTPases are essential for numerous cellular processes, and Rab8a is considered as a small GTPase that participates in the unconventional secretory pathway based on autophagy (Petkovic et al., 2014; Bel et al., 2017; Wall et al., 2017; Wall et al., 2018; Luo et al., 2018; Condon et al., 2018). Our previous study indicated that Rab8a was important for SAP secretion of AMs (Xu et al., 2022). Therefore, we rationally inferred that PAK4 may regulate SAP secretion by directly targeting Rab8a. In the present study, we found that the MiR-199a-3p inhibitors markedly suppressed Rab8a expression in LPS-induced macrophages, yet failed to inhibit SAP secretion after PAK4 inhibition, which indicated that PAK4 mediates SAP release *via* the Rab8a pathway.

## Conclusion

To sum up, our study suggests that the over-expression of MiR-199a-3p contributes to the progression of lung injury and inflammation in ARDS by promoting AM-derived SAP secretion mediated *via* the PAK4/Rab8a pathway. The suppression of endogenous MiR-199a-3p could provide pulmonary protection against LPS-induced ARDS. Thus, our results reveal that MiR-199a-3p could be considered as a latent candidate for the targeted molecular treatment of ARDS.

## Data availability statement

The raw data supporting the conclusions of this article will be made available by the authors, without undue reservation.

## Ethics statement

The animal study was reviewed and approved by the Committee of Animal Care and Use of Southeast University(Protocol number 20180106007).

## Author contributions

XX and XL performed the experiments, analyzed the data, and wrote the manuscript. XD took part in acquisition of the data. LL and YY designed and supervised this research. All authors contributed to the article and approved the submitted version.

## Funding

Our research was funded, in part, by National Natural Science Foundation of China (Grant numbers 81870066, 81670074, 81930058, 82270083, and 81971888), Six Talent Peaks Project in Jiangsu Province (Grant numbers TD-SWYY-003), and Social Development Specific Projects of Jiangsu Province (Grant number BE2020786).

## Acknowledgments

We thank all professors and colleagues working in the Jiangsu Provincial Key Laboratory of Critical Care Medicine.

## Conflict of interest

The authors declare that the research was conducted in the absence of any commercial or financial relationships that could be construed as a potential conflict of interest.

## Publisher’s note

All claims expressed in this article are solely those of the authors and do not necessarily represent those of their affiliated organizations, or those of the publisher, the editors and the reviewers. Any product that may be evaluated in this article, or claim that may be made by its manufacturer, is not guaranteed or endorsed by the publisher.
