# Supplementary material for: MiR-199a-3p-regulated alveolar macrophage-derived secretory autophagosomes exacerbate lipopolysaccharide-induced acute respiratory distress syndrome
Source: Front Cell Infect Microbiol. 2022 Nov 29;12:1061790. doi: 10.3389/fcimb.2022.1061790 (PMC9745060; doi:10.3389/fcimb.2022.1061790)
Supplement: Supplementary file 1 [file DataSheet_1.pdf]

Supplementary Figures

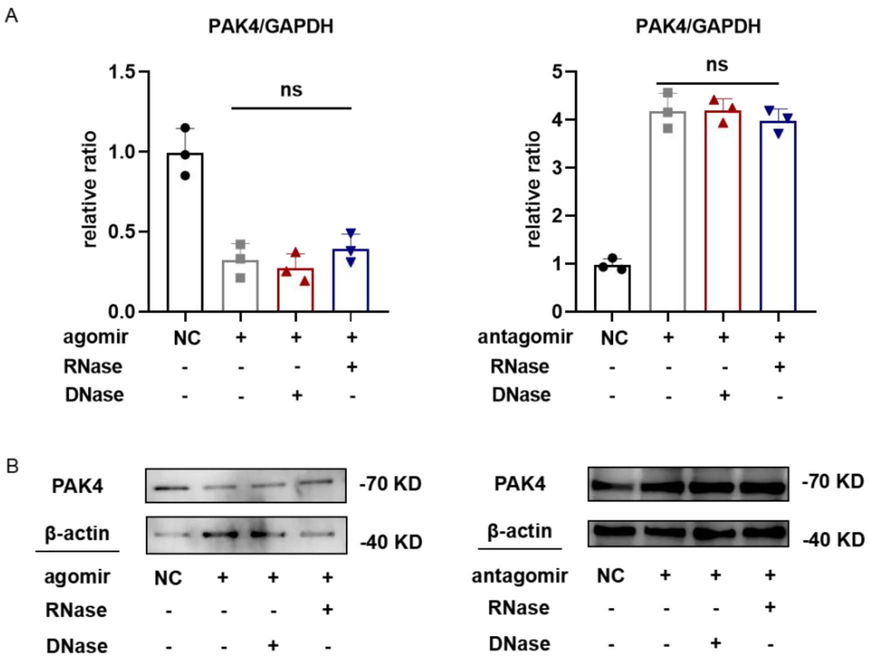

Figure S1. Neither RNase nor DNase administration affects the effect of agomir/antagomir on the expression of MiR-199a-3p target mRNA/protein in vitro. Agomir / antagomir of MiR-199a-3p was administered to RAW264.7 cells with or without RNase (20ug/ml) / DNase (1U/ml) administration. 24 hours later, the cells were harvested and evaluated for **(A)** target mRNA expression (RT-qPCR) and **(B)** protein expression (Western blotting) of PAK4 (n=3). Data were expressed as mean ± SD. NS, no significant difference, one-way ANOVA.

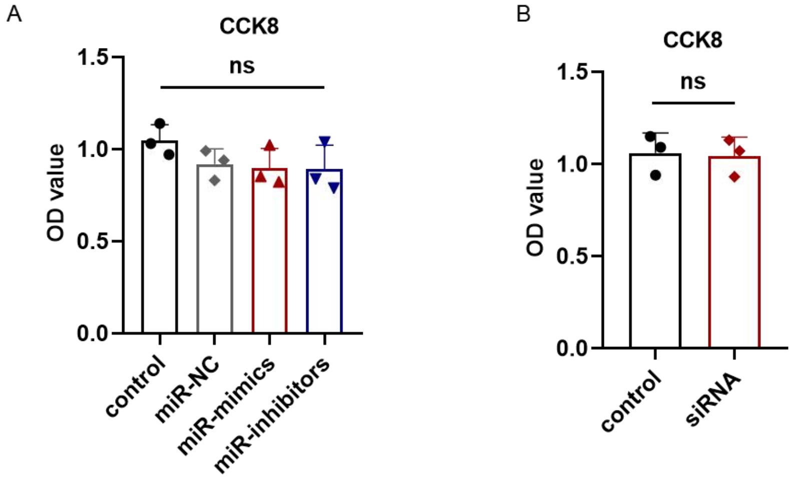

Figure S2. The cytotoxic influence of Cell transfection and RNA interference. **(A)** After transfection with MiR-199a-3p mimic, MiR-199a-3p inhibitor or negative controls, RAW264.7 cells were subjected to CCK-8 assay (n=3). **(B)** After transfection with siRNA, RAW264.7 cells were subjected to CCK-8 assay (n=3). Data were expressed as mean ± SD. NS,

no significant difference, Student' s t-test or one-way ANOVA.

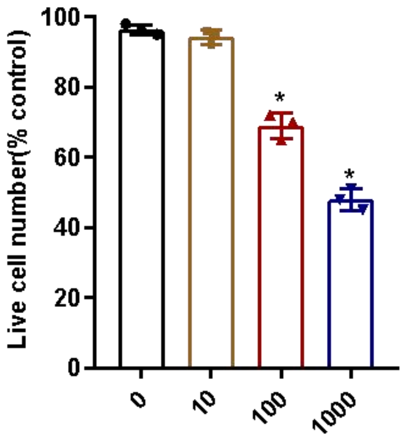

Figure S3. The effects of various concentrations of LPS on the viability of RAW264.7 cells. The cells were harvested and tested via trypan blue exclusion assay after LPS treatment for 24hours (n=3). Data were expressed as mean ± SD. **p*<0.05 vs. control group, one-way ANOVA.

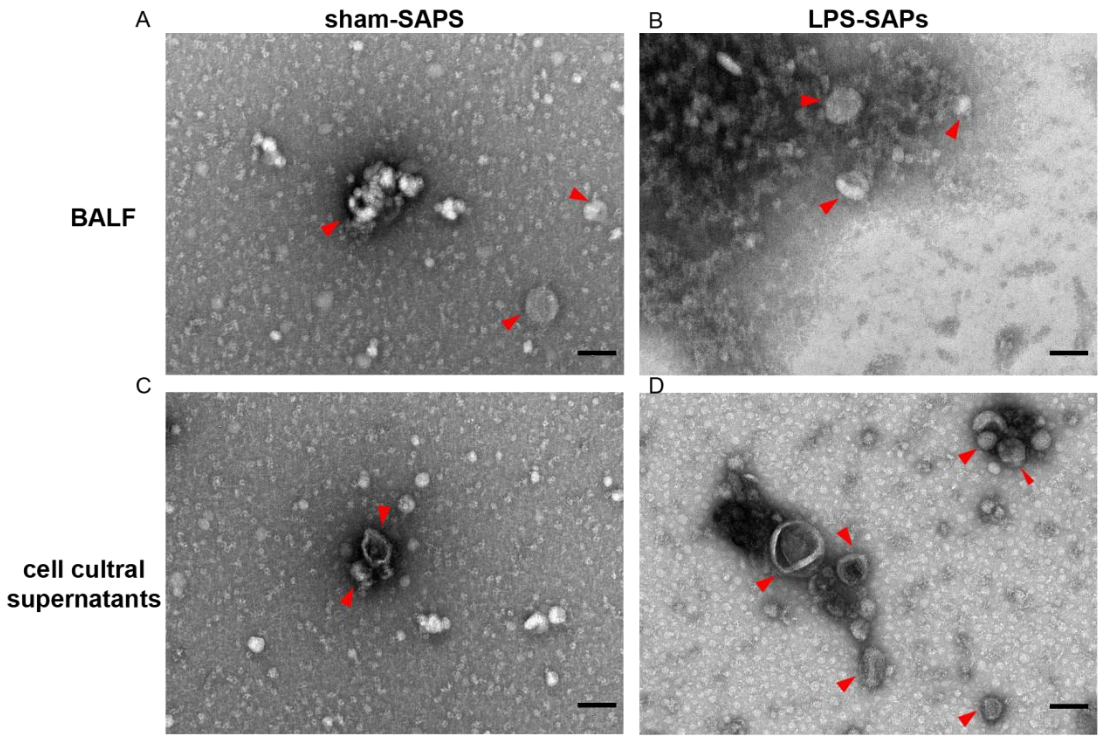

Figure S4. The morphology of SAPs isolated from BALF of mice and cell cultral supernantant were evaluated via transmission electron microscopy (TEM). **(A)** SAPs isolated from BALF of mice. **(B)** SAPs isolated from BALF of mice intratreally injected with LPS. **(C)** SAPs isolated from cell cultral supernantant of RAW264.7 cells. **(D)** SAPs isolated from cell cultral supernantant of RAW264.7 cells treated with LPS. SAPs are indicated by red arrow heads (Scale bar 200 nm).

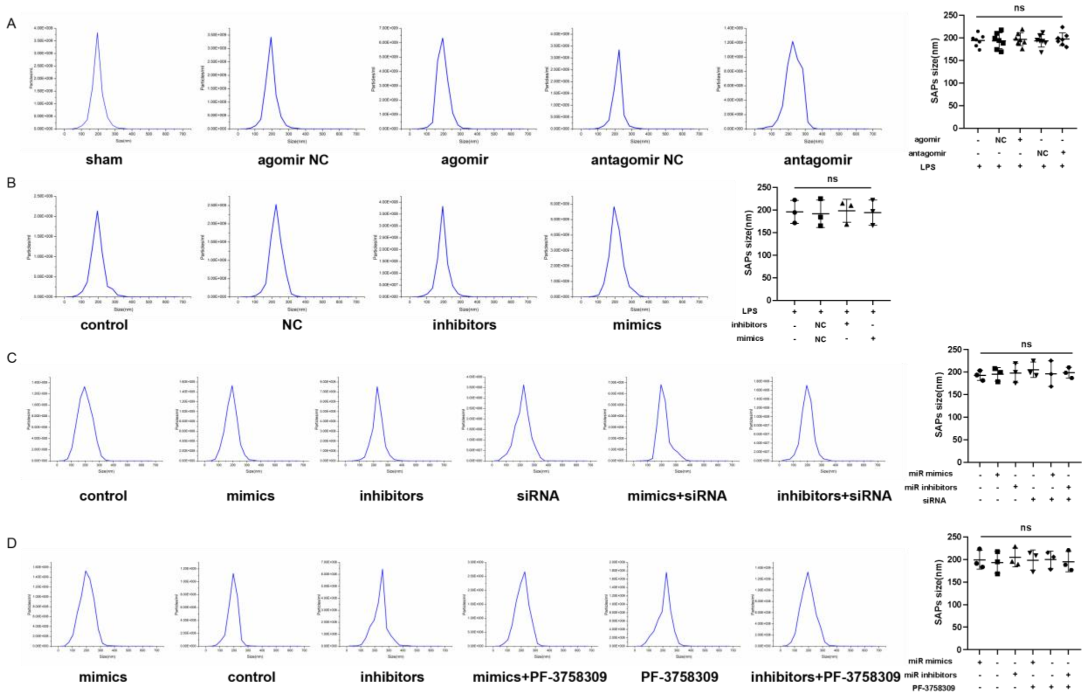

Figure S5. The size and concentration of the SAPs from BALF or cell cultural supernatants were assessed via NTA. (A) SAPs isolated from BALF from seven different mice of each group analysed by NTA in **figure 3D**. Left panel: NTA results obtained from one representative sample from each group of figure 3D. Right panel: comparison of SAPs sizes in each group indicating a mean peak particle size of approximately ~200 nm in diameter (n=7). **(B)** SAPs isolated from cell cultural supernatants from three different samples of each group analysed by NTA in **figure 4C**. Left panel: NTA results obtained from one representative sample from each group of figure 4C. Right panel: comparison of SAPs sizes in each group indicating a mean peak particle size of approximately ~200 nm in diameter (n=3). **(C)** SAPs isolated from cell cultural supernatants from three different samples in each group analysed by NTA in **figure 5C**. Left panel: NTA results obtained from one representative sample from each group of figure 5C. Right panel: comparison of SAPs sizes in each group indicating a mean peak particle size of approximately ~200 nm in diameter (n=3). **(D)** SAPs isolated from cell cultural supernatants from three different samples in each group analysed by NTA in **figure 6D**. Left panel: NTA results obtained from one representative sample from each group of figure 6D. Right panel: comparison of SAPs sizes in each group indicating a mean peak particle size of approximately ~200 nm in diameter (n=3). Data were expressed as mean ± SD. NS, no significant difference, one-way ANOVA.
